# Supplementary material for: The interactome of intact mitochondria by cross-linking mass spectrometry provides evidence for coexisting respiratory supercomplexes
Source: Mol Cell Proteomics. 2017 Dec 8;17(2):216–32. doi: 10.1074/mcp.RA117.000470 (PMC5795388; doi:10.1074/mcp.RA117.000470)
Supplement: Supplemental Data [file supp_RA117.000470_133922_0_supp_23978_fzffwf.zip › spectra_annotation/mito_DR_spectra_annotation/70-1-1-1-4-1.pdf]

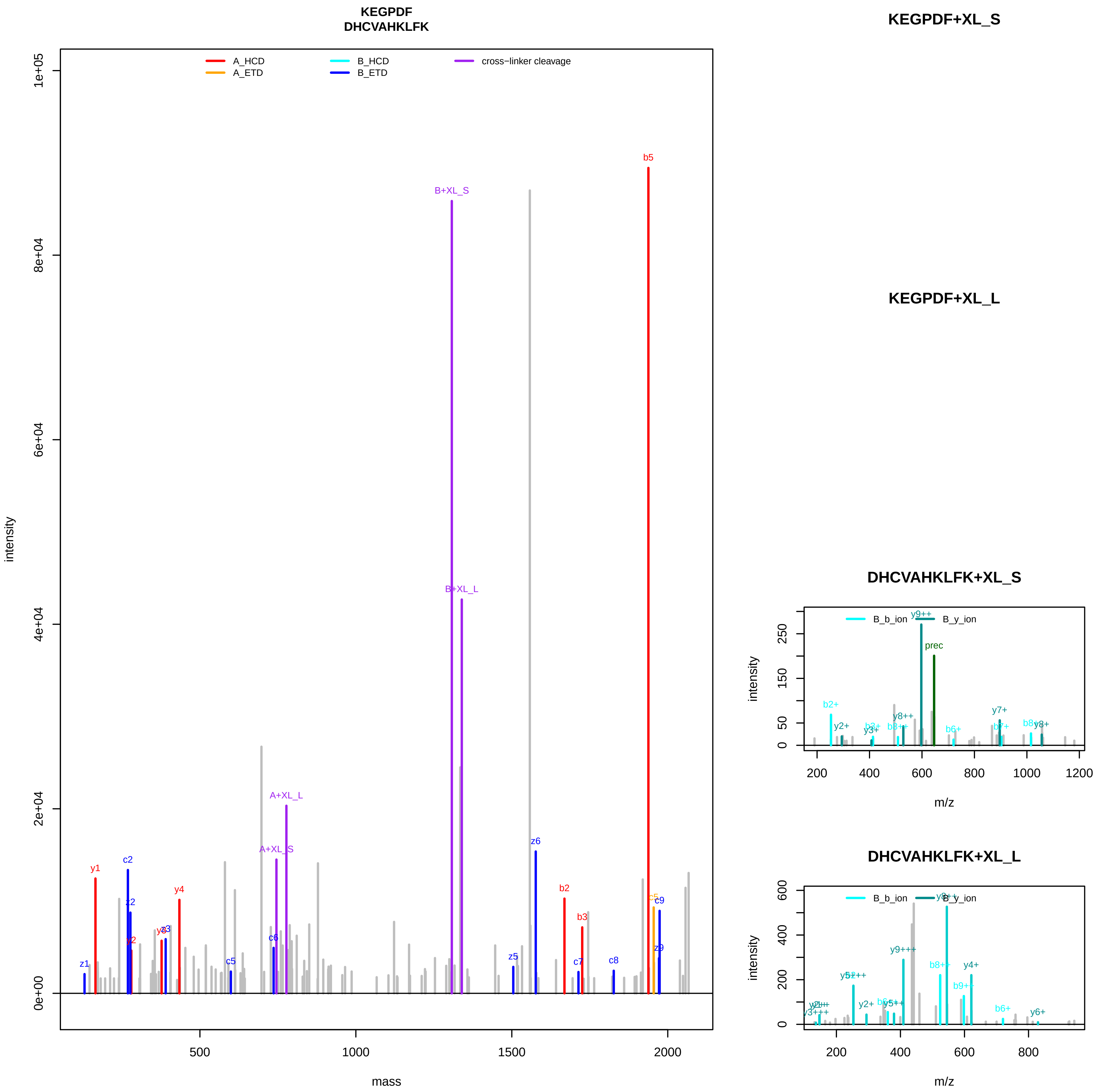
KEGPDF
DHCVAHKLFK
A_HCD
A_ETD
B_HCD
B_ETD
cross−linker cleavage
intensity
mass
B+XL_S
B+XL_L
A+XL_L
A+XL_S
b5
b2
b3
c5
c9
z9
c8
c7
z6
z5
c6
c5
y4
y3
z3
y2
z2
c2
y1
z1
1e+05
8e+04
6e+04
4e+04
2e+04
0e+00
500
1000
1500
2000
KEGPDF+XL_S
KEGPDF+XL_L
DHCVAHKLFK+XL_S
B_b_ion
B_y_ion
y9++
prec
b2+
y2+
y3+
y8++
b6+
y7+
y8+
m/z
DHCVAHKLFK+XL_L
y9+++
b8++
y4+
b9++
y2+
b6+
y6+
0
50
150
250
200
400
600
800
1000
1200
